# Vibration control of a nonlinear cantilever beam operating in the 3D space

**DOI:** 10.1038/s41598-022-16973-y

**Published:** 2022-08-15

**Authors:** Phuong-Tung Pham, Quoc Chi Nguyen, Mahnjung Yoon, Keum-Shik Hong

**Affiliations:** 1 Department of Mechatronics, Faculty of Mechanical Engineering, Ho Chi Minh City University of Technology (HCMUT), VNU-HCM, Ho Chi Minh City, Vietnam; 2 School of Mechanical Engineering, Pusan National University, Busan, 46241 South Korea; 3 POWER MnC Co., Ltd., Ulsan, 44988 South Korea

**Keywords:** Engineering, Mathematics and computing

## Abstract

This paper addresses a control problem of a nonlinear cantilever beam with translating base in the three-dimensional space, wherein the coupled nonlinear dynamics of the transverse, lateral, and longitudinal vibrations of the beam and the base’s motions are considered. The control scheme employs two control inputs applied to the beam’s base to control the base’s position while simultaneously suppressing the beam’s transverse, lateral, and longitudinal vibrations. According to the Hamilton principle, a hybrid model describing the nonlinear coupling dynamics of the beam and the base is established: This model consists of three partial differential equations representing the beam’s dynamics and two ordinary differential equations representing the base’s dynamics. Subsequently, the control laws are designed to move the base to the desired position and attenuate the beam’s vibrations in all three directions. The asymptotic stability of the closed-loop system is proven via the Lyapunov method. Finally, the effectiveness of the designed control scheme is illustrated via the simulation results.

## Introduction

The systems consisting of an elastic cantilever beam fixed on a translating base are found in various practical engineering applications, such as master fuel assemblies in nuclear refueling machines, robotic manipulators^1^, and micro-electro-mechanical systems^2–4^. In these systems, the base’s translational motion can produce large-amplitude vibrations of the beam in the three-dimensional (3D) space. This vibration becomes a significant negative factor in association with the system’s safety and performance. Therefore, it is necessary to analyze and control the 3D vibration of the beam, operated by a moving base, to ensure safety and performance.

**Figure 1:**
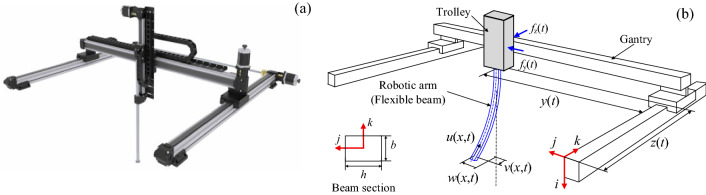
An example of nonlinear cantilever beams operating in the 3D space: (**a**) Gantry robot. (www.a-m-c.com/servo-drives-for-gantry-systems), (**b**) the defined coordinate system and motions.

The flexible cantilever beam is a distributed parameter system with an infinite number of vibration modes. Its dynamics are characterized by partial differential equations (PDEs)^5–8^. When a flexible beam is fixed on a translating base, the base’s dynamics (as a lumped parameter system) are described by ordinary differential equations (ODEs) to be considered simultaneously with the beam’s dynamics. Furthermore, if the amplitudes of the beam’s vibrations are large, 3D analysis of the beam’s dynamics should be performed; wherein the nonlinear coupling effects between the transverse, lateral, and longitudinal vibrations are considered, see Fig. 1.

The dynamic behaviors of the beam attached to a moving base have been studied in the literature^9–12^. Park et al. developed an equation of motion of the mass-beam-cart system, which is a beam with translating base, based on the Hamilton principle^13^. The system’s natural frequencies were also obtained by using modal analysis. Later, the vibration of a flexible beam fixed on a cart and carrying a moving mass was examined via an experimental study^14^. However, most researches on the beam attached to a translating base assume that the base moves along one direction, restricting the beam’s vibration to a two-dimensional space. In these works, only the transverse vibration was considered. For the beam with a translating base in the 3D space, Shah and Hong addressed the vibration problem of the master fuel assembly in nuclear refueling machines^15^. In their work, the nuclear fuel rod and the trolley, respectively, were treated as a flexible beam and a carrying base moving on the horizontal plane.

The control problem of distributed parameter systems, whose dynamics is described by PDEs, has been investigated in the literature^16–21^. Boundary control technique, wherein the control input is exerted to the PDE through its boundary conditions^22–25^, is a powerful tool for handling these systems. Contrary to the stationary beam, the vibration of the cantilever beam with a moving base can be suppressed via the control input applied at the base (i.e., the clamped end of the beam) or the beam’s tip. In this situation, we aim to simultaneously control of the base’s position and the beam’s vibration. These objectives can be achieved by either open-loop control^26,27^ or closed-loop control^15,28–33^: The input shaping control is the most feasible and practical open-loop control technique for beams with a moving base. In a study published by Shah et al.^26^, model parameters of an underwater system consisting of a beam and a translating base were determined using the model analysis method. Accordingly, the input shaping control law was designed to position the base and suppress the beam’s vibration. Pham et al.^27^ used the model parameters obtained through an experiment to design the input shaping control law for a non-uniform beam with a moving base. For the closed-loop control technique, Liu and Chao presented an experimental study on implementing the neuro-fuzzy approach to control a beam-cart system^28^. In their work, the piezoelectric transducers located at the beam’s tip were used to suppress the transverse vibration. Another closed-loop control of an Euler–Bernoulli beam with a translating base was done by Shah and Hong^15^, whereas the control of a Timoshenko beam attached to a moving base was presented by Pham et al.^34^.

Most studies on controlling the flexible beam attached to a translating base considered only the linear vibrations^15,26–28^. Under the assumption of small-amplitude vibration, the dynamic tension was ignored. However, in the case of large-amplitude vibrations, the negligence of the dynamic tension (which makes the beam’s dynamics nonlinear) can affect the system’s performance and stability. Also, the existing studies assumed that the beam’s vibration occurred in a plane. Thus, only the transverse or lateral vibrations were considered. Even though Shah and Hong^15^ investigate both the transverse and lateral vibrations of an Euler–Bernoulli beam, they ignored the coupled dynamics of the transverse and lateral vibrations; that is, the transverse vibration does not affect the time-evolution of the lateral vibration and vice versa.

Recently, the 3D vibration analysis of a beam has received significant attention^35–40^. Do and Pan^38^ and Do^39^ used the Euler–Bernoulli beam model with large-amplitude vibrations to model a flexible riser. The authors obtained a model describing the system’s transverse, lateral, and longitudinal vibrations. He et al.^41^, Ji and Liu^42,43^, and Liu et al.^44^ investigated the coupled dynamics of a 3D cantilever beam with a tip payload described by a set of PDEs and ODEs. Also, control problems of 3D beams, wherein the coupled dynamics of nonlinear vibrations, were investigated in the literature. However, these studies have either dealt with a beam attached to a stationary base or proposed control strategies wherein control forces/torques were applied to the beam’s tip. The implementation of control actions at the tip is not feasible or practical, see Fig. 1. It might be possible to put an actuator at the tip of a large cantilever beam of a space structure or a riser system. But, for gantry manipulators, surgeon robots, and flexible liquid handling robots, implementing the control forces/torques in the tip is not possible because the tip has to interact with an object or the environment.

The published papers in the literature on controlling cantilever-beam vibrations are restricted to: (i) The cases where the cantilever is affixed on a stationary base, and the free end has 3D motions^41–44^; and (ii) the beam is attached to a translating base, but the considered dynamics are linear by ignoring the coupled dynamics between the transversal and lateral vibrations of the beam^15,26–28^. Thus, the control problem of a nonlinear cantilever beam operating in the 3D space without using control input at the tip has not been solved yet.

In this paper, the beam’s longitudinal vibration and axial deformation (which makes the beam’s dynamics nonlinear) are further considered. In such cases of large-amplitude vibrations, the omission of longitudinal vibration and axial deformation can affect the system’s performance and lead to an erroneous result. Henthforth, the control problem of the nonlinear 3D vibrations of a beam affixed on a translating base without any additional actuators is addressed for the first time. The considered system is represented as a gantry robot consisting of the gantry, trolley, and flexible robotic arm depicted in Fig. 1. In the gantry robot, the gantry moves along the *k*-axis, whereas the trolley moves along the *j*-axis. A flexible robotic arm with a constant length is fixed to the trolley. The Hamilton principle is used to develop a novel hybrid model describing the nonlinear coupling dynamics of the robotic arm’s transverse, lateral, and longitudinal vibrations, and the rigid body motions of the gantry and trolley. Employing the Lyapunov method, boundary control laws are developed for simultaneous control of the trolley’s position, the gantry’s position, and the robotic arm’s 3D vibrations. The asymptotic stability of the closed-loop system is verified. Finally, the simulation results are provided.

The main contributions of this paper are summarized as follows: (i) A novel dynamic model of a flexible beam attached to a translating base, wherein the coupled dynamics of the nonlinear transverse, lateral, and longitudinal vibrations and the base’s motions are developed for the first time. (ii) A boundary control strategy using the control forces at the base for simultaneous position control and vibration suppression is designed. (iii) The asymptotic stability of the closed-loop system is proven by using the Lyapunov method, and simulation results are provided.

## Problem formulation

In Fig. 1, a flexible robotic arm is modeled as a uniform Euler–Bernoulli beam of length *l*. The motions of the gantry and trolley are generated by two control forces *f*_*z*_ and *f*_*y*_, respectively. The positions of the trolley and gantry are denoted by *y*(*t*) and *z*(*t*), respectively. The beam’s vibrations in the *i*, *j*, and *k* axes are defined as the longitudinal vibration *u*(*x*, *t*), the transverse vibration *w*(*x*, *t*), and the lateral vibration *v*(*x*, *t*), respectively. In this study, the subscripts *x* and *t*, i.e., (·)_*x*_ and (·)_*t*_, are the partial derivatives with respect to *x* and *t*, respectively, whereas $\dot{y}$ and $\dot{z}$ denotes the total derivative of *y*(*t*) and *z*(*t*) in *t*, respectively. The kinetic energy of the entire gantry, trolley, and beam system is given as follows:

1 $$K = \frac{1}{2}\rho A\int_{0}^{l} {\left[\left(y + w_{t} \right)^{2} + u_{t}^{2} + \left(z + v_{t} \right)^{2} \right]dx} + \frac{1}{2}\left(m_{{1}} + m_{{2}} \right)\dot{y}^{2} + \frac{1}{2}m_{{2}} \dot{z}^{2}$$

where *ρ* and *A* are the beam’s mass density and cross-sectional area; *m*_1_ and *m*_2_ are the gantry’s mass and trolley’s mass, respectively. The potential energy due to the axial force, the axial deformation, and the bending moment is given as follows:

2 $$U = \int_{0}^{l} {P(x)\left( {\frac{{w_{x}^{2} }}{2} + \frac{{v_{x}^{2} }}{2}} \right)dx} + \frac{1}{2}\int_{0}^{l} {EA\varepsilon (x,t)^{2} dx} + \frac{1}{2}EI_{y} \int_{0}^{l} {w_{xx}^{2} dx} + \frac{1}{2}EI_{z} \int_{0}^{l} {v_{xx}^{2} dx}$$

where $P(x) = \rho A(l - x)g$ is the axial force generated by the influence of the gravitational acceleration on the beam’s elements^45,46^, *E* denotes Young’s modulus, and *I*_*y*_ and *I*_*z*_ indicate the moments of inertia of the beam. The axial strain $\varepsilon (x,t)$ is given by the following approximation^47^:

3 $$\varepsilon (x,t) = u_{x} + w_{x}^{2} /2 + v_{x}^{2} /2.$$

The virtual work done on the system by the boundary control inputs and the friction is given as follows.

4 $$\delta W = f_{y} \delta y + f_{z} \delta z - c_{w} \int_{0}^{l} {w_{t} \delta wdx} - c_{u} \int_{0}^{l} {u_{t} \delta udx} - c_{v} \int_{0}^{l} {v_{t} \delta vdx}$$

where *c*_*w*_, *c*_*u*_, and *c*_*v*_ are the structral damping coefficients (i.e., the subscripts *w*, *u*, and *v* stand for transverse, longitudinal, and lateral, respectively). According to Hamilton’s principle, the dynamic model of the considered system and the corresponding boundary conditions are obtained as follows.

5 $$\rho A(\ddot{y} + w_{tt} ) + c_{w} w_{t} - (Pw_{x} )_{x} - EA[w_{x} (u_{x} + w_{x}^{2} /2 + v_{x}^{2} /2)]_{x} + EI_{y} w_{xxxx} = 0,$$

6 $$w(0,t) = w_{x} (0,t) = w_{xx} (l,t) = w_{xxx} (l,t) = 0,$$

7 $$\rho Au_{tt} + c_{u} u_{t} - EA[u_{x} + w_{x}^{2} /2 + v_{x}^{2} /2]_{x} = 0,$$

8 $$u(0,t) = u_{x} (l,t) + w_{x}^{2} (l,t)/2 + v_{x}^{2} (l,t)/2 = 0,$$

9 $$\rho A(\ddot{z} + v_{tt} ) + c_{v} v_{t} - (Pv_{x} )_{x} - EA[v_{x} (u_{x} + w_{x}^{2} /2 + v_{x}^{2} /2)]_{x} + EI_{z} v_{xxxx} = 0,$$

10 $$v(0,t) = v_{x} (0,t) = v_{xx} (l,t) = v_{xxx} (l,t) = 0,$$

11 $$(m_{{1}} + m_{{2}} )\ddot{y} - c_{w} \int_{0}^{l} {w_{t} dx} + EI_{y} w_{xxx} (0,t) = f_{y},$$

12 $$m_{2} \ddot{z} - c_{v} \int_{0}^{l} {v_{t} dx} + EI_{z} v_{xxx} (0,t) = f_{z}.$$

The dynamics of the considered system are represented by the nonlinear PDE-ODE model in (5)–(12): Eqs. (5)–(10) are PDEs describing the transverse, longitudinal, and lateral vibrations of the robotic arm, respectively, whereas the ODEs in (11) and (12) represent the dynamics of the gantry and the trolley, respectively. Observably, the beam’s motion affects the gantry and trolley’s motions and vice versa. Additionally, if the potential energy caused by the axial deformation is ignored (i.e., $\varepsilon^{2} = (u_{x} + w_{x}^{2} /2 + v_{x}^{2} /2)^{2} \cong 0$), the nonlinear terms in (5), (7), and (9) vanish. Then, the coupling dynamics between the transverse, lateral, and longitudinal vibrations can be decoupled.

## Controller design

The two control objectives are position control and vibration suppression: (i) Move the gantry and trolley carrying the flexible beam to the desired positions, and (ii) suppress the beam’s transverse, lateral, and longitudinal vibrations. In this paper, two forces *f*_*z*_ and *f*_*y*_ applied to the gantry and trolley are used as the control inputs to achieve the control objectives. The position errors of the trolley and gantry are defined as follows:

13 $$e_{y} = y - y_{{\text{d}}},$$

14 $$e_{z} = z - z_{{\text{d}}}$$

where *y*_d_ and *z*_d_ are the desired positions of the trolley and gantry, respectively. Based on the Lyapunov direct method, we design *f*_*z*_ and *f*_*y*_ to guarantee that the convergences of the vibrations, position errors, and velocities of the trolley and gantry to zero are achieved. The following control forces are proposed to stabilize the considered system.

15 $$f_{y} = - K_{1} \dot{y} - K_{2} e_{y} - K_{3} w_{xxx} (0,t),$$

16 $$f_{z} = - K_{4} \dot{z} - K_{5} e_{z} - K_{6} v_{xxx} (0,t)$$

where *K*_*i*_ (*i* = 1,2,…,6) are the control parameters. The implementation of these control laws requires the measurement of *w*_*xxx*_(0, *t*) and *v*_*xxx*_(0, *t*). In practice, these signals can be obtained by using strain gauge sensors attached at the clamped end of the beam.

The following lemmas and assumptions are used for stability analysis of the closed-loop system with the control laws given in (15) and (16).

### Lemma 1

^48^. *Let* $\varphi (x,t) \in {\mathbb{R}}$ *be a function defined on* $x \in [0,l]$ *and* $t \in [0,\infty )$ *that satisfies the boundary condition* $\varphi (0,t) = 0, \, \forall t \in [0,\infty )$*, the following inequalities hold.*

17 $$\int_{0}^{l} {\varphi^{2} (x,t)dx} \le l^{2} \int_{0}^{l} {\varphi_{x}^{2} (x,t)dx} , \, \forall x \in [0,l],$$

18 $$\varphi^{2} (x,t) \le l\int_{0}^{l} {\varphi_{x}^{2} (x,t)dx} , \, \forall x \in [0,l].$$

*Furthermore, if φ*(*x*, *t*) *satisfies φ*(0, *t*) = *φ*_*x*_(0, *t*) = 0, $\forall t \in [0,\infty ),$ *then the following inequalities hold*.

19 $$\int_{0}^{l} {\varphi_{x}^{2} (x,t)dx} \le l^{2} \int_{0}^{l} {\varphi_{xx}^{2} (x,t)dx} , \, \forall x \in [0,l],$$

20 $$\varphi^{2} (x,t) \le l^{3} \int_{0}^{l} {\varphi_{xx}^{2} (x,t)dx} , \, \forall x \in [0,l].$$

### Lemma 2

^49^. *Let* $\varphi_{1} (x,t),\varphi_{2} (x,t) \in {\mathbb{R}}$ *be a function defined on* $x \in [0,l]$*. Then, the following inequality holds.*

21 $$\varphi_{1} (x,t)\varphi_{2} (x,t) \le \varphi_{1}^{2} (x,t)/\delta + \delta \varphi_{2}^{2} (x,t), \, \forall \delta > 0.$$

### Lemma 3

^50^. *If φ*(*x*, *t*): [0, *l*] × ${\mathbb{R}}^{ + } \to {\mathbb{R}}$ *is uniformly bounded,* $\{ \varphi (x,t)\}_{x \in [0,l]}$ *is equicontinuous on t, and* $\mathop {\lim }\limits_{t \to \infty } \int_{0}^{t} {\left\| {\varphi (x,\tau )} \right\|^{2} d\tau }$ *exists and is finite, then* $\mathop {\lim }\limits_{t \to \infty } \left\| {\varphi (x,t)} \right\| = 0$.

### Assumption 1

^21^. *The transverse vibration w*(*x*, *t*), *the lateral vibration v*(*x*, *t*), *and the longitudinal vibration u*(*x*, *t*) *of a flexible beam satisfy the following inequalities*: $u_{x}^{2} \le w_{x}^{2} /2$ *and* $u_{x}^{2} \le v_{x}^{2} /2$. *By using Lemma **1**, we obtain.*

22 $$\int_{0}^{l} {u^{2} dx} \le l^{2} \int_{0}^{l} {u_{x}^{2} dx} \le \frac{{l^{2} }}{4}\int_{0}^{l} {w_{x}^{2} dx} + \frac{{l^{2} }}{4}\int_{0}^{l} {v_{x}^{2} dx} \le \frac{{l^{4} }}{4}\int_{0}^{l} {w_{xx}^{2} dx} + \frac{{l^{4} }}{4}\int_{0}^{l} {v_{xx}^{2} dx} .$$

### Assumption 2

^51^. *If the potential energy of the system in* (2) *is bounded for* $\forall t \in [0,\infty )$, *then* $w_{xx} (x,t)$, $w_{xxx} (x,t)$, $v_{xx} (x,t)$, *and* $v_{xxx} (x,t)$ *are bounded for* $\forall t \in [0,\infty )$.

Based on the system’s mechanical energy, the following Lyapunov function candidate is introduced:

23 $$V = V_{0} + V_{1}$$

where

24 $$\begin{gathered} V_{0} = \frac{\rho A}{2}\left[ {\int_{0}^{l} {(\dot{y} + w_{t} )^{2} dx} + \int_{0}^{l} {(\dot{z} + v_{t} )^{2} dx} + \int_{0}^{l} {u_{t}^{2} dx} } \right] + \left( {1 + \frac{{2\alpha_{2} }}{\rho A}} \right)\left[ {\int_{0}^{l} {P\left( {\frac{{w_{x}^{2} }}{2} + \frac{{v_{x}^{2} }}{2}} \right)dx} } \right. \\ \left. { + \frac{1}{2}EA\int_{0}^{l} {\left( {u_{x} + \frac{{w_{x}^{2} }}{2} + \frac{{v_{x}^{2} }}{2}} \right)^{2} dx} + EI_{y} \int_{0}^{l} {w_{xx}^{2} dx} + EI_{z} \int_{0}^{l} {v_{xx}^{2} dx} } \right] + \frac{1}{2}(m_{1} + m_{2} )\dot{y}^{2} \\ + \frac{1}{2}m_{2} \dot{z}^{2} + \frac{1}{2}\alpha_{1} e_{y}^{2} + \alpha_{2} \int_{0}^{l} {(w_{t}^{2} + u_{t}^{2} + v_{t}^{2} )dx} + \frac{1}{2}\alpha_{3} e_{z}^{2} , \\ \end{gathered}$$

25 $$\begin{gathered} V_{1} = \rho A\beta_{1} \int_{0}^{l} {ww_{t} dx} + \frac{1}{2}\beta_{1} c_{w} \int_{0}^{l} {w^{2} dx} + \rho A\beta_{2} \int_{0}^{l} {uu_{t} dx} + \frac{1}{2}\beta_{2} c_{u} \int_{0}^{l} {u^{2} dx} + (\beta_{3} c_{w} /\rho A)\int_{0}^{l} {w\dot{y}dx} \\ + \beta_{3} \int_{0}^{l} {\dot{y}(\dot{y} + w_{t} )dx} + \beta_{4} \dot{y}e_{y} + \beta_{5} \int_{0}^{l} {e_{y} (\dot{y} + w_{t} )dx} + \rho A\beta_{6} \int_{0}^{l} {vv_{t} dx} + \frac{1}{2}\beta_{6} c_{v} \int_{0}^{l} {v^{2} dx} \\ + (\beta_{7} c_{v} /\rho A)\int_{0}^{l} {v\dot{z}dx} + \beta_{7} \int_{0}^{l} {\dot{z}(\dot{z} + v_{t} )dx} + \beta_{8} \dot{z}e_{z} + \beta_{9} \int_{0}^{l} {e_{z} (\dot{z} + v_{t} )} dx \\ \end{gathered}$$

where *α*_*i*_ (*i* = 1, 2, 3) and *β*_*j*_ (*j* = 1, 2, …, 9) are positive coefficients.

### Lemma 4

*The Lyapunov function candidate in* (23) *is upper and lower bounded as follows*.

26 $$0 \le \lambda_{1} W_{1} \le V \le \lambda_{2} W_{2}$$

*where* $\lambda_{1}$ *and* $\lambda_{2}$ *are positive constants, and*

27 $$W_{1} = \dot{y}^{2} + \dot{z}^{2} + \int_{0}^{l} {w_{t}^{2} dx} + \int_{0}^{l} {u_{t}^{2} dx} + \int_{0}^{l} {v_{t}^{2} dx} + \int_{0}^{l} {w_{xx}^{2} dx} + \int_{0}^{l} {v_{xx}^{2} dx} + e_{y}^{2} + e_{z}^{2} ,$$

28 $$\begin{gathered} W_{2} = \dot{y}^{2} + \dot{z}^{2} + \int_{0}^{l} {w_{t}^{2} dx} + \int_{0}^{l} {u_{t}^{2} dx} + \int_{0}^{l} {v_{t}^{2} dx} + \int_{0}^{l} {P(w_{x}^{2} + v_{x}^{2} )dx} + \int_{0}^{l} {(u_{x} + w_{x}^{2} /2 + v_{x}^{2} /2)^{2} dx} \\ + \int_{0}^{l} {w_{xx}^{2} dx} + \int_{0}^{l} {v_{xx}^{2} dx} + e_{y}^{2} + e_{z}^{2} . \\ \end{gathered}$$

Proof of Lemma 4: See Appendix A.

### Lemma 5

*Under the control laws* (15) *and* (16), *the time derivative of the Lyapunov function candidate in* (23) *is upper bounded as follows*.

29 $$\dot{V} \le - \lambda V$$

*where λ is a positive constant.*

Proof of Lemma 5: See Appendix B.

### Theorem 1.

*Consider a hybrid system described by* (5)-(12) *under control laws* (15–16) *and Assumptions 1 and 2*. *Control parameters K*_*i*_ (*i* = 1, 2, …, 6) *are selected to satisfy the conditions in* (A.15)–(A.23), (B.9), (B.17)–(B.22), *and* (B.25)–(B.35). *The asymptotic stability of the closed-loop system in the sense that the transverse vibration w*(*x*, *t*), *lateral vibration v*(*x*, *t*), *longitudinal vibration u*(*x*, *t*), *and position errors* (13) *and* (14) *converge to zero is guaranteed. Additionally, the control laws are bounded.*

Proof of Theorem: Lemma 4 reveals that the Lyapunov function candidate in (23) is a positive definite. According to Lemma 5, we obtain

30 $$V(t) \le e^{ - \lambda t} V(0) \le V(0) < \infty$$

We define the norm of a spatiotemporal function as follows: $\left\| {w(x,t)} \right\| = \left( {\int_{0}^{l} {w^{2} (x,t)dx} } \right)^{1/2}$. Using Lemmas 1 and 4 and Assumption 1, the following inequalities are obtained.

31 $$w^{2} (x,t) \le l^{3} \int_{0}^{l} {w_{xx}^{2} (x,t)dx} \le l^{3} W_{1} \le l^{3} V/\lambda_{1} < \infty,$$

32 $$v^{2} (x,t) \le l^{3} \int_{0}^{l} {v_{xx}^{2} (x,t)dx} \le l^{3} W_{1} \le l^{3} V/\lambda_{1} < \infty,$$

33 $$u^{2} (x,t) \le l\int_{0}^{l} {u_{x}^{2} (x,t)dx} \le \frac{1}{2}l\int_{0}^{l} {w_{x}^{2} (x,t)dx} \le \frac{1}{2}l^{3} \int_{0}^{l} {w_{xx}^{2} (x,t)dx} \le \frac{1}{2}l^{3} V\lambda_{1} < \infty ,$$

34 $$e_{y}^{2} (t) \le W_{1} \le V/\lambda_{1} < \infty,$$

35 $$\dot{e}_{y}^{2} (t) \le W_{1} \le V/\lambda_{1} < \infty,$$

36 $$e_{z}^{2} (t) \le W_{1} \le V/\lambda_{1} < \infty,$$

37 $$\dot{e}_{z}^{2} (t) \le W_{1} \le V/\lambda_{1} < \infty.$$

Inequalities (31–37) assure *w*(*x*, *t*), *u*(*x*, *t*), *v*(*x*, *t*), $e_{y}$, $\dot{e}_{y}$, $e_{z}$, and $\dot{e}_{z}$ are all uniformly bounded. Similarly, we also obtain the boundedness of $\left\| {w(x,t)} \right\|^{2}$, $\left\| {w_{t} (x,t)} \right\|^{2}$, $\left\| {u(x,t)} \right\|^{2}$, $\left\| {u_{t} (x,t)} \right\|^{2}$, $\left\| {v(x,t)} \right\|^{2}$, and $\left\| {v_{t} (x,t)} \right\|^{2}$ based on Lemmas 4 and 5.

$$- \left\| {w(x,t)} \right\|^{2} \ge - l^{4} W_{1} \ge - l^{4} V/\lambda_{1} \ge l^{4} \dot{V}/\lambda \lambda_{1}$$

38 $$\Rightarrow \mathop {\lim }\limits_{t \to \infty } \int_{0}^{t} {\left\| {w(x,\tau )} \right\|^{2} d\tau } \le - l^{4} \mathop {\lim }\limits_{t \to \infty } \left( {V(t) - V(0)} \right)/\lambda \lambda_{1} < \infty,$$

$$- \left\| {v(x,t)} \right\|^{2} \ge - l^{4} W_{1} \ge - l^{4} V/\lambda_{1} \ge l^{4} \dot{V}/\lambda \lambda_{1}$$

39 $$\Rightarrow \mathop {\lim }\limits_{t \to \infty } \int_{0}^{t} {\left\| {v(x,\tau )} \right\|^{2} d\tau } \le - l^{4} \mathop {\lim }\limits_{t \to \infty } \left( {V(t) - V(0)} \right)/\lambda \lambda_{1} < \infty,$$

$$- \left\| {u(x,t)} \right\|^{2} \ge - l^{2} \int_{0}^{l} {u_{x}^{2} (x,t)dx} \ge - \frac{1}{2}l^{2} \int_{0}^{l} {w_{x}^{2} (x,t)dx} \ge - \frac{1}{2}l^{4} W_{1} \ge - \frac{1}{2}l^{4} V\lambda_{1} \ge \frac{1}{2}l^{4} \dot{V}\lambda \lambda_{1}$$

40 $$\Rightarrow \mathop {\lim }\limits_{t \to \infty } \int_{0}^{t} {\left\| {u(x,\tau )} \right\|^{2} d\tau } \le - \frac{1}{2}l^{4} \mathop {\lim }\limits_{t \to \infty } \left( {V(t) - V(0)} \right)\lambda \lambda_{1} < \infty.$$

Additionally, the following results also imply that *w*(*x*, *t*), *u*(*x*, *t*), and *v*(*x*, *t*) are equicontinuous in *t*.

41 $$d\left\| {w(x,t)} \right\|^{2} /dt = 2\int_{0}^{l} {w(x,t)w_{t} (x,t)dx} \le \left\| {w(x,t)} \right\|^{2} + \left\| {w_{t} (x,t)} \right\|^{2} < \infty ,$$

42 $$d\left\| {v(x,t)} \right\|^{2} /dt = 2\int_{0}^{l} {v(x,t)v_{t} (x,t)dx} \le \left\| {v(x,t)} \right\|^{2} + \left\| {v_{t} (x,t)} \right\|^{2} < \infty ,$$

43 $$d\left\| {u(x,t)} \right\|^{2} /dt = 2\int_{0}^{l} {u(x,t)u_{t} (x,t)dx} \le \left\| {u(x,t)} \right\|^{2} + \left\| {u_{t} (x,t)} \right\|^{2} < \infty .$$

Accordingly, we can conclude that $\mathop {\lim }\limits_{t \to \infty } \left\| {w(x,t)} \right\| = 0,$ $\mathop {\lim }\limits_{t \to \infty } \left\| {v(x,t)} \right\| = 0$, and $\mathop {\lim }\limits_{t \to \infty } \left\| {u(x,t)} \right\| = 0$ via Lemma 3.

Furthermore, Lemmas 4 and 5 also imply

44 $$- e_{y}^{2} (t) \ge - W_{1} (t) \ge - V(t)/\lambda_{1} \ge \dot{V}(t)/\lambda_{1} \lambda \Rightarrow \mathop {\lim }\limits_{t \to \infty } \int_{0}^{t} {e_{y}^{2} (\tau )d\tau } \le - \mathop {\lim }\limits_{t \to \infty } \left( {V(t) - V(0)} \right)/\lambda_{1} \lambda < \infty,$$

45 $$- e_{z}^{2} (t) \ge - W_{1} (t) \ge - V(t)/\lambda_{1} \ge \dot{V}(t)/\lambda_{1} \lambda \Rightarrow \mathop {\lim }\limits_{t \to \infty } \int_{0}^{t} {e_{z}^{2} (\tau )} d\tau \le - \mathop {\lim }\limits_{t \to \infty } \left( {V(t) - V(0)} \right)/\lambda_{1} \lambda < \infty.$$

Based on Barbalat’s Lemma, we can conclude that $\mathop {\lim }\limits_{t \to \infty } \left| {e_{y} } \right| = 0$ and $\mathop {\lim }\limits_{t \to \infty } \left| {e_{z} } \right| = 0$.

Inequality (30) implies the boundedness of *V*(*t*). It follows that the potential energy function is also a bounded function. Under Assumption 2, *w*_*xx*_(*x*, *t*), *w*_*xxx*_(*x*, *t*), *v*_*xx*_(*x*, *t*), and *v*_*xxx*_(*x*, *t*) are bounded. Inequalities (34)-(37) reveal that $e_{y}$, $\dot{e}_{y}$,$e_{z}$, and $\dot{e}_{z}$ are also bounded. Finally, we can conclude that the control laws in (15) and (16) are bounded. Theorem 1 is proved.

## Simulation results

In this section, numerical simulations are performed to illustrate the effectiveness of the proposed control laws. The system parameters used in the numerical simulation are shown in Table 1. According to these system parameters, the control gains in (15) and (16) are selected as *K*_1_ = 750, *K*_2_ = 950, *K*_3_ = 1.12 × 10^4^, *K*_4_ = 550, *K*_5_ = 750, and *K*_6_ = 2.34 × 10^4^. Control parameters *K*_*i*_ (*i* = 1, 2,…, 6) are calculated based on design parameters *k*_*i*_, *α*_*n*_, *β*_*j*_, and *δ*_*k*_ (*n* = 1, 2, 3; *j* = 1, 2,…, 7; *k* = 1, 2,…, 9). These design parameters have been selected to satisfy the conditions in (A.15–A.23), (B.9), (B.17–B.22), and (B.25–B.35). Some parameters, such as *δ*_1_, *δ*_2_, *δ*_4_, and *α*_2_, can be pre-determined based on the necessary conditions of (A.15–A.17) and (A.19). By substituting these parameters into (B.9), (B.17), and (B.20), *β*_1_, *β*_3_, *β*_6_, *β*_7_, *k*_3_, and *k*_6_ are calculated. Then, the ranges of *β*_2_, *δ*_0_, *δ*_3_, *β*_4_, *β*_8_, *β*_5_, *β*_9_, *δ*_5_, *δ*_6_, *δ*_7_, and *δ*_8_ can be determined in turn based on (A.21–A.22), (B.19), (B.22), and (B.30)-(B.35). We substitute (B.18) into (A.22) and choose large enough values of *k*_1_ and *k*_2_ such that (A.22) and (B.25–B.26) hold. Similarly, we substitute (B.18) into (A.23) and select *k*_4_ and *k*_5_ to satisfy (A.23) and (B.28–B.29). Finally, *α*_1_ and *α*_3_ are calculated using (B.18) and (B.21).

**Table 1:** System parameters.

Parameter	Definition	Value
*l*	Beam length	1.5 m
*h*	Beam height	0.01 m
*b*	Beam width	0.006 m
*A*	Beam’s cross-section area	0.6 × 10^–4^ m^2^
*I*_*y*_	Beam’s initial moment	1.8 × 10^–10^ m^4^
*I*_*z*_	Beam’s initial moment	5 × 10^–10^ m^4^
*ρ*	Beam’s mass density	2700 kg/m^2^
*E*	Young’s modulus	69 $\times$ 10^9^ Pa
*m*_1_	Gantry’s mass	30 kg
*m*_2_	Trolley’s mass	20 kg
*c*_*w*_	Transverse damping coefficient	0.05 Ns/m
*c*_*v*_	Lateral damping coefficient	0.05 Ns/m
*c*_*u*_	Longitudinal damping coefficient	0.05 Ns/m
*y*_d_	Trolley’s desired position	6 m
*z*_d_	Gantry’s desired position	4 m

The simulations were performed by using MATLAB, wherein the finite difference method was utilized to determine the approximate solutions for the equations of motion. The approximate solutions’ accuracy and simulation speed depend on the sizes of the time and space steps (i.e., Δ*t* and Δ*x*, respectively). By using a large time step size, approximate solutions of PDEs are determined quickly. However, a too-large time step size reduces the accuracy of the solution and further leads to instability. Contrarily, the quality of the solutions can be improved by selecting a smaller step size. In this case, the simulation duration increases significantly. Therefore, selecting appropriate step sizes is necessary to guarantee a balance between accuracy and simulation speed. In this paper, the time and space step sizes are selected as follows: Δ*t* = 10^–5^ and Δ*x* = 0.075*.*

The dynamic behavior of the proposed control law (15) and (16) is compared with two typical cases: (i) Using the traditional PD control law and (ii) using the zero-vibration (ZV) input shaping control. For the input shaping control, the ZV input shapers are designed based on the cantilever beam’s natural frequencies and damping ratios. The natural frequencies are determined via the solution of the frequency Eq.^26^, whereas the damping ratios are calculated by using the logarithmic decrement algorithm^27^.

**Figure 2:**
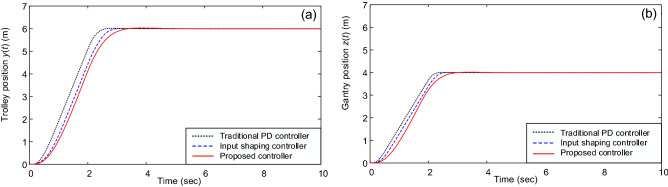
(**a**) Trolley’s position and (**b**) gantry’s position.

**Figure 3:**
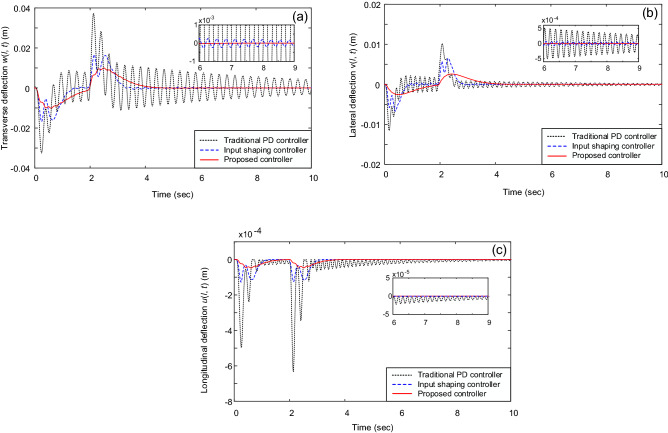
Vibrations of the beam’s tip: (**a**) Transverse vibration *w*(*l*, *t*), (**b**) lateral vibration *v*(*l*, *t*), and (**c**) longitudinal vibration *u*(*l*, *t*).

**Figure 4:**
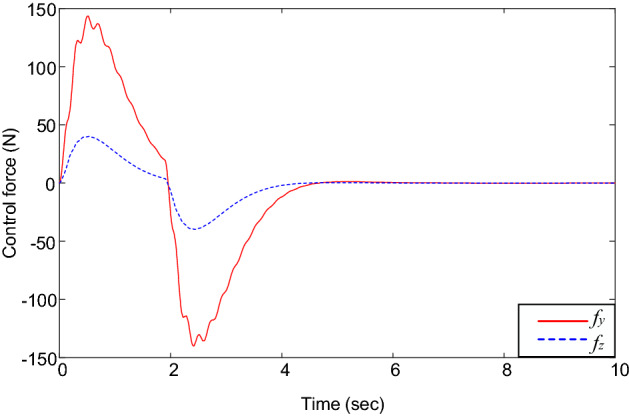
Control forces under the proposed control law.

**Figure 5:**
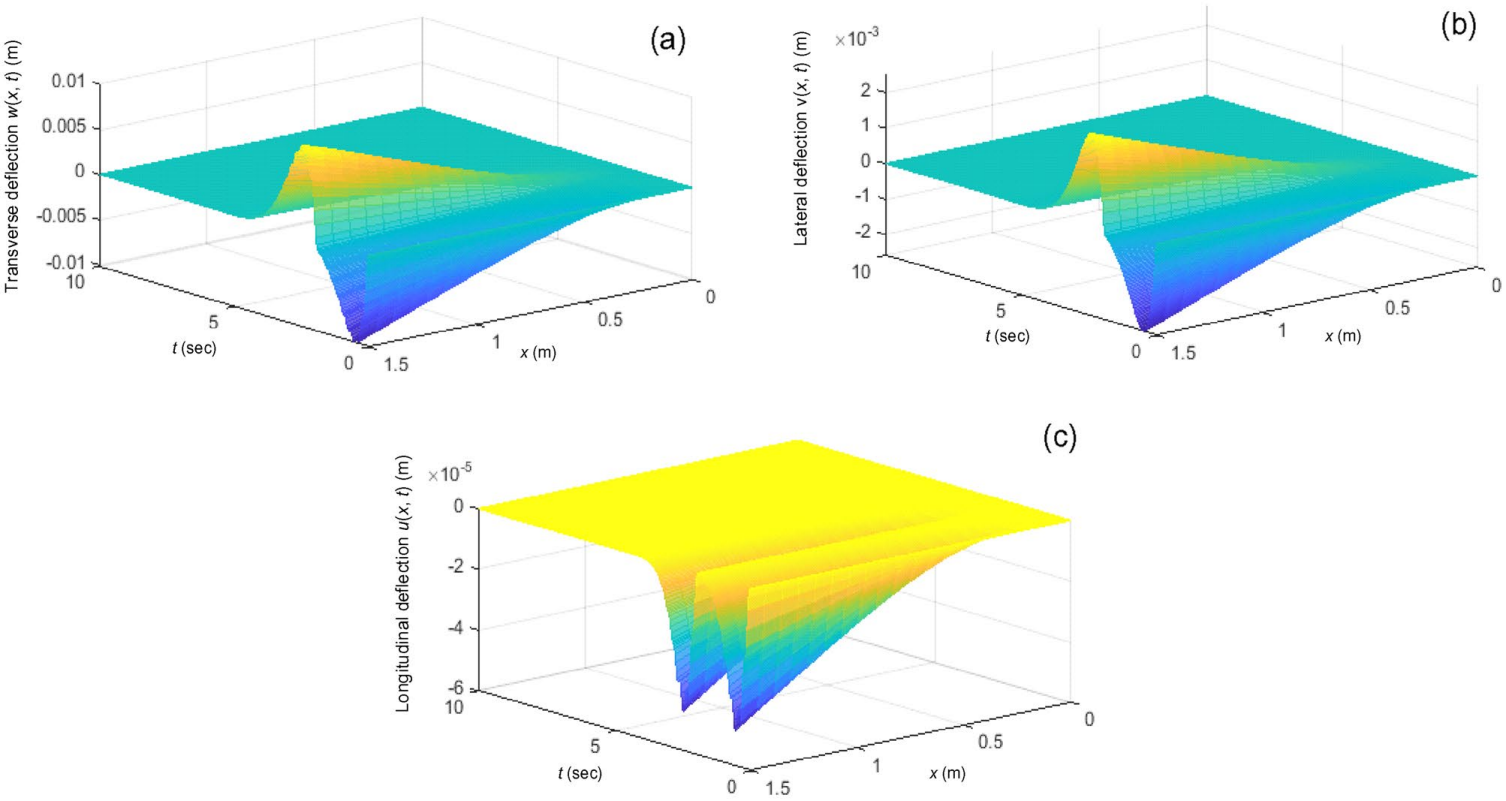
Vibrations of the three-dimensional flexible beam under the proposed control law: (**a**) Transverse vibration *w*(*x*, *t*), (**b**) lateral vibration *v*(*x*, *t*), and (**c**) longitudinal vibration *u*(*x*, *t*).

Figures 2 and 3 illustrate the system’s responses under different controllers. Figure 2 shows the trolley’s position and gantry’s position, whereas Fig. 3 reveal the vibrations of the beam’s tip. It shows that the PD controller, input shaping controller, and the proposed controller can move the trolley and gantry to the desired position (i.e., Fig. 2). However, the traditional PD controller cannot deal with the beam’s vibrations, see Fig. 3. In this case, vibration suppression was done only based on structural damping; therefore, it requires a significant amount of time. Contrary to the PD control, the system’s vibrations under the input shaping control and the proposed control law were quickly suppressed, see Fig. 3. Most tip oscillations were eliminated when the trolley and gantry reached the desired positions (i.e., at *t* ≈ 4 s). Furthermore, the proposed control law showed an outstanding vibration suppression capability compared with the input shaping control (i.e., see the magnified graphs in Fig. 3). The control forces under the proposed control law and suppression of the three vibrations are depicted in Figs. 4 and 5.

**Figure 6:**
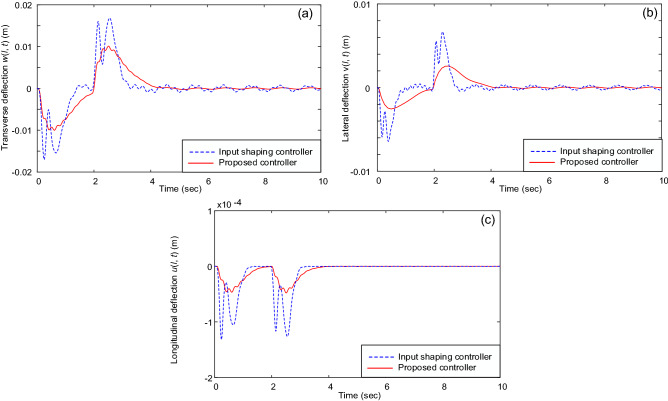
Vibrations of the beam’s tip under boundary disturbances: (**a**) Transverse vibration *w*(*l*, *t*), (**b**) lateral vibration *v*(*l*, *t*), and (**c**) longitudinal vibration *u*(*l*, *t*).

**Figure 7:**
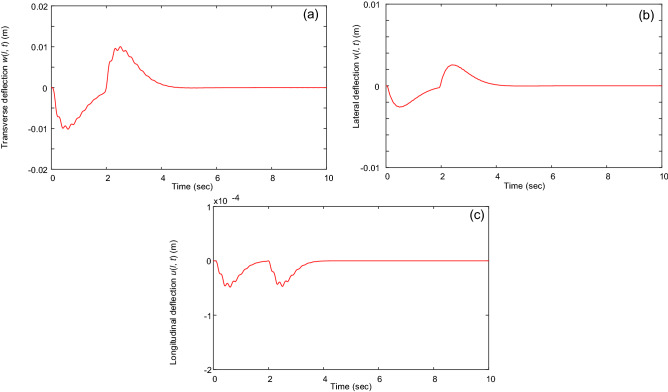
Vibrations of the beam’s tip under measurement noises in feedback signals: (**a**) Transverse vibration *w*(*l*, *t*), (**b**) lateral vibration *v*(*l*, *t*), and (**c**) longitudinal vibration *u*(*l*, *t*).

Figures 6 and 7 reveal the robustness of the proposed control law. In Fig. 6, we consider the system under the influence of disturbances. Two boundary disturbances, *d*_*y*_(*t*) = 10sin(20π*t*) and *d*_*z*_(*t*) = 8sin(20π*t*), are applied to the trolley and gantry, respectively. As shown in Fig. 6, the proposed control law can still eliminate most of the vibrations of the beam system under boundary disturbance. The sensitivity of the proposed control law to the measurement noises of the sensors is considered in Fig. 7. In this case, 20% noises are added in the feedback signals *w*_*xxx*_(0, *t*) and *v*_*xxx*_(0, *t*). Observably, the measurement noises have no significant effects on the responses of the closed-loop system under the proposed control law. The simulation results show that the proposed control law is not too sensitive to disturbances and measurement noises.

## Conclusions

This paper investigated a vibration suppression problem of the three-dimensional cantilever beam fixed on a translating base. The equations of motions describing the nonlinear coupling dynamics of the beam’s transverse, lateral, longitudinal vibrations, the gantry, and the trolley were developed using the Hamilton principle. Accordingly, the control laws were designed. The asymptotic stability of the closed-loop system in the sense that the beam’s transverse vibration, lateral vibration, longitudinal vibration, and gantry’s position error and trolley’s position error converge to zero was proven via the Lyapunov method. Simulation results showed the effectiveness of the proposed control laws. In practical gantry systems, the length of the robotic arm varies in time, and the system is subjected to disturbances. Our future work will address extending the current control strategy to a varying-length flexible beam with moving base, providing experimental results.

## Data Availability

The data and codes generated or analyzed in this paper can be available upon the communication with the corresponding author.

## Appendix A

The proof of Lemma 4 is shown in here. By using Lemmas 1–2, we obtain

A.1 $$\left| {\int_{0}^{l} {ww_{t} dx} } \right| \le (l^{4} /\delta_{0} )\int_{0}^{l} {w_{xx}^{2} dx} + \delta_{0} \int_{0}^{l} {w_{t}^{2} dx} ,$$

A.2 $$\left| {\int_{0}^{l} {uu_{t} dx} } \right| \le \frac{1}{4}l^{4} \delta_{1} \left( {\int_{0}^{l} {w_{xx}^{2} dx} + \int_{0}^{l} {v_{xx}^{2} dx} } \right) + \delta_{1} \int_{0}^{l} {u_{t}^{2} dx} ,$$

A.3 $$\left| {\int_{0}^{l} {w\dot{y}dx} } \right| \le l^{4} \int_{0}^{l} {w_{xx}^{2} dx} + l\dot{y}^{2} ,$$

A.4 $$\left| {\int_{0}^{l} {\dot{y}w_{t} dx} } \right| \le (l/\delta_{2} )\dot{y}^{2} + \delta_{2} \int_{0}^{l} {w_{t}^{2} dx} ,$$

A.5 $$\left| {\dot{y}e_{y} } \right| \le \dot{y}^{2} + e_{y}^{2} ,$$

A.6 $$\left| {\int_{0}^{l} {e_{y} (\dot{y} + w_{t} )dx} } \right| \le 2le_{y}^{2} + l\dot{y}^{2} + \int_{0}^{l} {w_{t}^{2} dx} ,$$

A.7 $$\left| {\int_{0}^{l} {vv_{t} dx} } \right| \le (l^{4} /\delta_{3} )\int_{0}^{l} {v_{xx}^{2} dx} + \delta_{3} \int_{0}^{l} {v_{t}^{2} dx} ,$$

A.8 $$\left| {\int_{0}^{l} {v\dot{z}dx} } \right| \le l^{4} \int_{0}^{l} {v_{xx}^{2} dx} + l\dot{z}^{2} ,$$

A.9 $$\left| {\int_{0}^{l} {\dot{z}v_{t} dx} } \right| \le (l/\delta_{4} )\dot{z}^{2} + \delta_{4} \int_{0}^{l} {v_{t}^{2} dx} ,$$

A.10 $$\left| {\dot{z}e_{z} } \right| \le \dot{z}^{2} + e_{z}^{2} ,$$

A.11 $$\left| {\int_{0}^{l} {e_{z} (\dot{z} + v_{t} )} dx} \right| \le 2le_{z}^{2} + l\dot{z}^{2} + \int_{0}^{l} {v_{t}^{2} dx} .$$

According to (A.1)-(A.11), the lower bound of *V*_0_ and *V*_1_ are given by

A.12 $$\begin{gathered} V_{0} \ge \frac{1}{2}\rho A\int_{0}^{l} {u_{t}^{2} dx} + \left( {\frac{1}{2} + \alpha_{2} /\rho A} \right)\left[ {EI_{y} \int_{0}^{l} {w_{xx}^{2} dx} } \right.\left. { + EI_{z} \int_{0}^{l} {v_{xx}^{2} dx} } \right] \\ + \frac{1}{2}(m_{1} + m_{2} )\dot{y}^{2} + \frac{1}{2}m_{2} \dot{z}^{2} + \frac{1}{2}\alpha_{1} e_{y}^{2} + \frac{1}{2}\alpha_{3} e_{z}^{2} \\ + \alpha_{2} \int_{0}^{l} {w_{t}^{2} dx} + \alpha_{2} \int_{0}^{l} {u_{t}^{2} dx} + \alpha_{2} \int_{0}^{l} {v_{t}^{2} dx} , \\ \end{gathered}$$

A.13 $$\begin{gathered} V_{1} \ge \left[ {\beta_{3} l(1 - c_{w} /\rho A - 1/\delta_{2} ) - \beta_{4} - \beta_{5} l} \right]\dot{y}^{2} \\ + \left[ {\beta_{7} l(1 - c_{v} /\rho A} \right.\left. { - 1/\delta_{4} ) - \beta_{8} - \beta_{9} l} \right]\dot{z}^{2} \\ - \left( {\rho A\beta_{1} \delta_{1} + \beta_{3} \delta_{2} + \beta_{5} } \right)\int_{0}^{l} {w_{t}^{2} dx} - \rho A\delta_{1} \beta_{2} \int_{0}^{l} {u_{t}^{2} dx} - \left( {\rho A\beta_{6} \delta_{3} + \beta_{7} \delta_{4} + \beta_{9} } \right)\int_{0}^{l} {v_{t}^{2} } dx \\ - l^{4} \left( {\rho A\beta_{1} /\delta_{1} + \rho A\beta_{2} /4\delta_{1} + \beta_{3} c_{w} /\rho A} \right)\int_{0}^{l} {w_{xx}^{2} dx} \\ - l^{4} \left( {\rho A\beta_{6} /\delta_{3} } \right.\left. { + \beta_{7} c_{v} /\rho A + \rho A\beta_{2} /4\delta_{1} } \right)\int_{0}^{l} {v_{xx}^{2} dx} \\ - \left( {\beta_{4} + 2\beta_{5} l} \right)e_{y}^{2} - \left( {\beta_{8} + 2\beta_{9} l} \right)e_{z}^{2} . \\ \end{gathered}$$

Based on (A.12) and (A.13), the lower bound of the Lyapunov function candidate is obtained as follows.

A.14 $$\begin{aligned} V & \ge \left[ {(m_{1} + m_{2} )/2 + \beta_{3} l\left( {1 - c_{w} /\rho A - 1/\delta_{2} } \right) - \beta_{4} - \beta_{5} l} \right]\dot{y}^{2} \\ & + \left[ {m_{2} /2 + \beta_{7} l\left( {1 - c_{v} /\rho A - 1/\delta_{4} } \right) - \beta_{8} - \beta_{9} l} \right]\dot{z}^{2} \\& \quad + \left[ {\alpha_{2} } \right. - \left. {\left( {\rho A\beta_{1} \delta_{0} + \beta_{3} \delta_{2} + \beta_{5} } \right)} \right]\int_{0}^{l} {w_{t}^{2} } dx + \left[ {\alpha_{2} + \rho A/2} \right.\left. { - \delta_{1} \rho A\beta_{2} } \right]\int_{0}^{l} {u_{t}^{2} dx} \\ & + \left[ {\alpha_{2} - \left( {\rho A\beta_{6} \delta_{3} + \beta_{7} \delta_{4} + \beta_{9} } \right)} \right]\int_{0}^{l} {v_{t}^{2} dx} \\ & + \left[ {EI_{y} \left( {1/2 + \alpha_{2} /\rho A} \right) - l^{4} \left( {\rho A\beta_{1} /\delta_{0} + \rho A\beta_{2} /4\delta_{1} } \right.} \right.\left. {\left. { + \beta_{3} c_{w} /\rho A} \right)} \right]\int_{0}^{l} {w_{xx}^{2} dx} \\& + \left[ {EI_{z} \left( {1/2 + \alpha_{2} /\rho A} \right) - l^{4} \left( {\rho A\beta_{6} /\delta_{3} } \right.} \right.\left. {\left. { + \rho A\beta_{2} /4\delta_{1} + \beta_{7} c_{v} /\rho A} \right)} \right]\int_{0}^{l} {v_{xx}^{2} dx} \\& + \left[ {\alpha_{1} /2 - \left( {\beta_{4} + 2\beta_{5} l} \right)} \right]e_{y}^{2} + \left[ {\alpha_{5} /2 - \left( {\beta_{8} + 2\beta_{9} l} \right)} \right]e_{z}^{2} \\ &\ge \lambda_{1} W_{1} \\ \end{aligned}$$

where $\lambda_{1} = \min \left( {\lambda_{11} ,\lambda_{12} ,...,\lambda_{19} } \right)$ and $\alpha_{i}$,$\beta_{j}$, and $\delta_{k}$ ($i = 1,2,...,6$, $j = 1,2,...,9$, and $k = 1,2,...,4$) are selected to guarantee that $\lambda_{1n}$ ($i = 1,2,...,9$) satisfy

A.15 $$\lambda_{11} = (m_{1} + m_{2} )/2 + \beta_{3} l\left( {1 - c_{w} /\rho A - 1/\delta_{2} } \right) - \beta_{4} - \beta_{5} l > 0,$$

A.16 $$\lambda_{12} = m_{2} /2 + \beta_{7} l\left( {1 - c_{v} /\rho A - 1/\delta_{4} } \right) - \beta_{8} - \beta_{9} l > 0,$$

A.17 $$\lambda_{13} = \alpha_{2} - \left( {\rho A\beta_{1} \delta_{0} + \beta_{3} \delta_{2} + \beta_{5} } \right) > 0,$$

A.18 $$\lambda_{14} = \alpha_{2} + \rho A/2 - \delta_{1} \rho A\beta_{2} > 0,$$

A.19 $$\lambda_{15} = \alpha_{2} - \left( {\rho A\beta_{6} \delta_{3} + \beta_{7} \delta_{4} + \beta_{9} } \right) > 0,$$

A.20 $$\lambda_{16} = EI_{y} (1/2\left. { + \alpha_{2} /\rho A} \right) - l^{4} \left( {\rho A\beta_{1} /\delta_{0} + \rho A\beta_{2} /4\delta_{1} + \beta_{3} c_{w} /\rho A} \right) > 0,$$

A.21 $$\lambda_{17} = EI_{z} \left( {1/2 + \alpha_{2} /\rho A} \right) - l^{4} \left( {\rho A\beta_{6} /\delta_{3} + \rho A\beta_{2} /4\delta_{1} + \beta_{7} c_{v} /\rho A} \right) > 0,$$

A.22 $$\lambda_{18} = \alpha_{1} /2 - \left( {\beta_{4} + 2\beta_{5} l} \right) > 0,$$

A.23 $$\lambda_{19} = \alpha_{5} /2 - \left( {\beta_{8} + 2\beta_{9} l} \right) > 0.$$

Similarly, the upper bound of the Lyapunov function candidate can be obtained by using (A.1)-(A.11), that is

A.24 $$\begin{aligned} V & \le \left[ {\rho Al + (m_{1} + m_{2} )/2 + \beta_{4} + \beta_{5} l + \beta_{3} l\left( {1 + c_{w} /\rho A + 1/\delta_{2} } \right)} \right]\dot{y}^{2} \\ &+ \left[ {\rho Al + m_{2} /2 + \beta_{8} + \beta_{9} l + \beta_{7} l\left( {1 + c_{v} /\rho A + 1/\delta_{4} } \right)} \right]\dot{z}^{2} \\ &+ \left( {\rho A + \rho A\beta_{1} \delta_{0} + \beta_{3} \delta_{2} + \beta_{5} + \alpha_{2} } \right)\int_{0}^{l} {w_{t}^{2} dx} + \left( {\rho A + \rho A\beta_{6} \delta_{3} } \right.\left. { + \beta_{7} \delta_{4} + \beta_{9} + \alpha_{2} } \right)\int_{0}^{l} {v_{t}^{2} dx} \\ &+ \left( {\rho A/2 + \delta_{1} \rho A\beta_{2} + \alpha_{2} } \right)\int_{0}^{l} {u_{t}^{2} dx} \\ &+ \left( {1/2 + \alpha_{2} /\rho A} \right)\int_{0}^{l} {P(w_{x}^{2} + v_{x}^{2} )dx} + \left( {1/2 + \alpha_{2} /\rho A} \right)EA\int_{0}^{l} {(u_{x} + w_{x}^{2} /2 + v_{x}^{2} /2)^{2} dx} \\ &+ \left[ {EI_{y} \left( {1/2 + \alpha_{2} /\rho A} \right)} \right. + l^{4} \left( {\rho A\beta_{1} /\delta_{0} + \beta_{1} c_{w} /2 + \rho A\beta_{2} /4\delta_{1} + \beta_{2} c_{w} /8} \right.\left. {\left. { + \beta_{3} c_{w} /\rho A} \right)} \right]\int_{0}^{l} {w_{xx}^{2} dx} \\ &+ \left[ {EI_{z} \left( {1/2 + \alpha_{2} /\rho A} \right) + l^{4} \left( {\rho A\beta_{6} /\delta_{3} } \right.} \right.\left. {\left. { + \beta_{6} c_{v} /2 + \rho A\beta_{2} /4\delta_{1} + \beta_{2} c_{v} /8 + \beta_{7} c_{v} /\rho A} \right)} \right]\int_{0}^{l} {v_{xx}^{2} dx} \\ &+ \left( {\alpha_{1} /2 + \beta_{4} + 2\beta_{5} l} \right)e_{y}^{2} + \left( {\alpha_{3} /2 + \beta_{8} + 2\beta_{9} l} \right)e_{z}^{2} \\ & \le \lambda_{2} W_{2} \\ \end{aligned}$$

where $$\lambda_{2} = \max \left( {\lambda_{21} ,\lambda_{22} ,...,\lambda_{211} } \right)$$ and

A.25 $$\lambda_{21} = \rho Al + (m_{1} + m_{2} )/2 + \beta_{4} + \beta_{5} l + \beta_{3} l\left( {1 + c_{w} /\rho A + 1/\delta_{2} } \right),$$

A.26 $$\lambda_{22} = \rho Al + m_{2} /2 + \beta_{8} + \beta_{9} l + \beta_{7} l\left( {1 + c_{v} /\rho A + 1/\delta_{4} } \right),$$

A.27 $$\lambda_{23} = \rho A + \rho A\beta_{1} \delta_{0} + \beta_{3} \delta_{2} + \beta_{5} + \alpha_{2},$$

A.28 $$\lambda_{24} = \rho A + \rho A\beta_{6} \delta_{3} + \beta_{7} \delta_{4} + \beta_{9} + \alpha_{2},$$

A.29 $$\lambda_{25} = \rho A/2 + \delta_{1} \rho A\beta_{2} + \alpha_{2},$$

A.30 $$\lambda_{26} = 1/2 + \alpha_{2} /\rho A,$$

A.31 $$\lambda_{27} = \left( {1/2 + \alpha_{2} /\rho A} \right)EA,$$

A.32 $$\lambda_{28} = EI_{y} \left( {1/2 + \alpha_{2} /\rho A} \right) + \left. {l^{4} \left( {\rho A\beta_{1} /\delta_{0} + \beta_{1} c_{w} /2 + \rho A\beta_{2} /4\delta_{1} + \beta_{2} c_{w} /8} \right. + \beta_{3} c_{w} /\rho A} \right),$$

A.33 $$\lambda_{29} = EI_{z} \left( {1/2 + \alpha_{2} /\rho A} \right) + l^{4} \left( {\rho A\beta_{6} /\delta_{3} } \right.\left. { + \beta_{6} c_{v} /2 + \rho A\beta_{2} /4\delta_{1} + \beta_{2} c_{v} /8 + \beta_{7} c_{v} /\rho A} \right),$$

A.34 $$\lambda_{210} = \alpha_{1} /2 + \beta_{4} + 2\beta_{5} l,$$

A.35 $$\lambda_{211} = \alpha_{3} /2 + \beta_{8} + 2\beta_{9} l.$$

Based on (A.14–A.23) and (A.24–A.25), Lemma 4 is proven.

## Appendix B

The proof of Lemma 5 is shown here. The time derivative of *V*_0_ is derived as follows.

B.1 $$\begin{aligned} \dot{V}_{0} &= \rho A\int_{0}^{l} {(\dot{y} + w_{t} )(\ddot{y} + w_{tt} )dx} + \rho A\int_{0}^{l} {(\dot{z} + v_{t} )(\ddot{z} + v_{tt} )dx} + \rho A\int_{0}^{l} {u_{t} u_{tt} dx} \\ &+ \left( {1 + 2\alpha_{2} /\rho A} \right)\left[ {\int_{0}^{l} {P\left( {w_{x} w_{xt} + v_{x} v_{xt} } \right)dx} } \right. + EA\int_{0}^{l} {\left( {u_{x} + w_{x}^{2} /2 + v_{x}^{2} /2} \right)\left( {u_{xt} + w_{x} w_{xt} + v_{x} v_{xt} } \right)dx} \\ &+ EI_{y} \int_{0}^{l} {w_{xx} w_{xxt} dx} + \left. {EI_{z} \int_{0}^{l} {v_{xx} v_{xxt} dx} } \right] + (m_{1} + m_{2} )\dot{y}\ddot{y} + m_{2} \dot{z}\ddot{z} + \alpha_{1} e_{y} \dot{y} + \alpha_{3} e_{z} \dot{z} \\ &+ 2\alpha_{2} \left( {\int_{0}^{l} {w_{t} w_{tt} dx} + \int_{0}^{l} {u_{t} u_{tt} dx} + \int_{0}^{l} {v_{t} v_{tt} dx} } \right). \\ \end{aligned}$$

For notational convenience, *ε* is used instead of $$(u_{x} + w_{x}^{2} /2$$$$+ v_{x}^{2} /2)$$ (i.e., *ε* is the axial strain given in (3)). Substituting the dynamic model in (5)-(12) into (B.1) yields

B.2 $$\begin{aligned} \dot{V}_{0} &= - c_{w} \int_{0}^{l} {w_{t}^{2} dx} - c_{u} \int_{0}^{l} {u_{t}^{2} dx} - c_{v} \int_{0}^{l} {v_{t}^{2} dx} - c_{w} \int_{0}^{l} {\dot{y}w_{t} dx} - c_{v} \int_{0}^{l} {\dot{z}v_{t} dx} \\ &+ \left. {\left[ {Pw_{x} \dot{y}} \right]} \right|_{0}^{l} + \left. {\left[ {Pv_{x} \dot{z}} \right]} \right|_{0}^{l} + \left. {\left[ {EA\varepsilon w_{x} \dot{y}} \right]} \right|_{0}^{l} + \left. {\left[ {EA\varepsilon v_{x} \dot{z}} \right]} \right|_{0}^{l} - \left. {\left[ {EI_{y} \dot{y}w_{xxx} } \right]} \right|_{0}^{l} - \left. {\left[ {EI_{z} \dot{z}v_{xxx} } \right]} \right|_{0}^{l} \\ &+ \left( {1 + 2\alpha_{2} /\rho A} \right)\left\{ {\left. {\left[ {Pw_{x} w_{t} } \right]} \right|_{0}^{l} } \right. + \left. {\left[ {Pv_{x} v_{t} } \right]} \right|_{0}^{l} + \left. {\left[ {EA\varepsilon u_{t} } \right]} \right|_{0}^{l} + \left. {\left[ {EA\varepsilon w_{x} w_{t} } \right]} \right|_{0}^{l} + \left. {\left[ {EA\varepsilon v_{x} v_{t} } \right]} \right|_{0}^{l} \\ &+ EI_{y} \left. {\left[ {w_{xx} w_{xt} - w_{t} w_{xxx} } \right]} \right|_{0}^{l} \left. { + EI_{z} \left. {\left[ {v_{xx} v_{xt} - v_{t} v_{xxx} } \right]} \right|_{0}^{l} } \right\} + (m_{1} + m_{2} )\dot{y}\ddot{y} + m_{2} \dot{z}\ddot{z} \\ &- (2c_{w} \alpha_{2} /\rho A)\int_{0}^{l} {w_{t}^{2} dx} - (2c_{u} \alpha_{2} /\rho A)\int_{0}^{l} {u_{t}^{2} dx} - (2c_{v} \alpha_{2} /\rho A)\int_{0}^{l} {v_{t}^{2} dx} \\ &- 2\alpha_{2} \ddot{y}\int_{0}^{l} {w_{t} dx} - 2\alpha_{2} \ddot{z}\int_{0}^{l} {v_{t} dx} + \alpha_{1} e_{y} \dot{y} + \alpha_{3} e_{z} \dot{z}. \\ \end{aligned}$$

By using the boundary conditions and $P(l) = 0$, (B.2) can be rewritten as

$$\begin{aligned} \dot{V}_{0} &= - c_{w} \int_{0}^{l} {w_{t}^{2} dx} - c_{u} \int_{0}^{l} {u_{t}^{2} dx} - c_{v} \int_{0}^{l} {v_{t}^{2} dx} - \dot{y}c_{w} \int_{0}^{l} {w_{t} dx} - \dot{z}c_{v} \int_{0}^{l} {v_{t} dx} + EI_{y} \dot{y}w_{xxx} (0,t) + EI_{z} \dot{z}v_{xxx} (0,t) \\ &+ (m_{1} + m_{2} )\dot{y}\ddot{y} + m_{2} \dot{z}\ddot{z} - (2c_{w} \alpha_{2} /\rho A)\int_{0}^{l} {w_{t}^{2} dx} - (2c_{u} \alpha_{2} /\rho A)\int_{0}^{l} {u_{t}^{2} dx} - (2c_{v} \alpha_{2} /\rho A)\int_{0}^{l} {v_{t}^{2} dx} \\ &- 2\alpha_{2} \ddot{y}\int_{0}^{l} {w_{t} dx} - 2\alpha_{2} \ddot{z}\int_{0}^{l} {v_{t} dx} + \alpha_{1} e_{y} \dot{y} + \alpha_{3} e_{z} \dot{z} \\ \end{aligned}$$

B.3 $$\begin{aligned} &= - c_{w} \left( {1 + 2\alpha_{2} /\rho A} \right)\int_{0}^{l} {w_{t}^{2} dx} - c_{u} \left( {1 + 2\alpha_{2} /\rho A} \right)\int_{0}^{l} {u_{t}^{2} dx} - c_{v} \left( {1 + 2\alpha_{2} /\rho A} \right)\int_{0}^{l} {v_{t}^{2} dx} + \dot{y}f_{y} + \dot{z}f_{z} \\ &- 2\alpha_{2} \ddot{y}\int_{0}^{l} {w_{t} dx} - 2\alpha_{2} \ddot{z}\int_{0}^{l} {v_{t} dx} + \alpha_{1} e_{y} \dot{y} + \alpha_{3} e_{z} \dot{z}. \\ \end{aligned}$$

The time derivative of *V*_1_ is derived as follows.

B.4 $$\begin{aligned} \dot{V}_{1} &= \rho A\beta_{1} \int_{0}^{l} {w_{t}^{2} dx} + \rho A\beta_{1} \int_{0}^{l} {ww_{tt} dx} + \beta_{1} c_{w} \int_{0}^{l} {ww_{t} dx} + \rho A\beta_{2} \int_{0}^{l} {u_{t}^{2} dx} + \rho A\beta_{2} \int_{0}^{l} {uu_{tt} dx} + \beta_{2} c_{u} \int_{0}^{l} {uu_{t} dx} \\ &+ (\beta_{3} c_{w} /\rho A)\int_{0}^{l} {w_{t} \dot{y}dx} + (\beta_{3} c_{w} /\rho A)\int_{0}^{l} {w\ddot{y}dx} + \beta_{3} \int_{0}^{l} {\ddot{y}(\dot{y} + w_{t} )dx} + \beta_{3} \int_{0}^{l} {\dot{y}(\ddot{y} + w_{tt} )dx} + \beta_{4} \ddot{y}e_{y} \\ &+ \beta_{4} \dot{y}^{2} + \beta_{5} \int_{0}^{l} {\dot{y}(\dot{y} + w_{t} )dx} + \beta_{5} \int_{0}^{l} {e_{y} (\ddot{y} + w_{tt} )dx} + \rho A\beta_{6} \int_{0}^{l} {v_{t}^{2} dx} + \rho A\beta_{6} \int_{0}^{l} {vv_{tt} dx} + \beta_{6} c_{v} \int_{0}^{l} {vv_{t} dx} \\ &+ (\beta_{7} c_{v} /\rho A)\int_{0}^{l} {v_{t} \dot{z}dx} + (\beta_{7} c_{v} /\rho A)\int_{0}^{l} {v\ddot{z}dx} + \beta_{7} \int_{0}^{l} {\ddot{z}(\dot{z} + v_{t} )dx} + \beta_{7} \int_{0}^{l} {\dot{z}(\ddot{z} + v_{tt} )dx} + \beta_{8} \ddot{z}e_{z} \\ &+ \beta_{8} \dot{z}^{2} + \beta_{9} \int_{0}^{l} {\dot{z}(\dot{z} + v_{t} )dx} + \beta_{9} \int_{0}^{l} {e_{z} (\ddot{z} + v_{tt} )dx} . \\ \end{aligned}$$

Using the dynamic model and boundary conditions in (5)-(12) yields

B.5 $$\begin{aligned} \dot{V}_{1} &= \left( {\beta_{4} + \beta_{5} l} \right)\dot{y}^{2} + \left( {\beta_{8} + \beta_{9} l} \right)\dot{z}^{2} + \rho A\beta_{1} \int_{0}^{l} {w_{t}^{2} dx} + \rho A\beta_{2} \int_{0}^{l} {u_{t}^{2} dx} + \rho A\beta_{6} \int_{0}^{l} {v_{t}^{2} dx} \\ &+ \beta_{1} \int_{0}^{l} {w\left( {Pw_{x} } \right)_{x} dx} + \beta_{1} \int_{0}^{l} {w\left( {EA\varepsilon w_{x} } \right)_{x} dx} + \beta_{2} \int_{0}^{l} {EAu\varepsilon_{x} dx} + \beta_{6} \int_{0}^{l} {v\left( {Pv_{x} } \right)_{x} dx} \\ &+ \beta_{6} \int_{0}^{l} {v\left( {EA\varepsilon v_{x} } \right)_{x} dx} - EI_{y} \beta_{1} \int_{0}^{l} {ww_{xxxx} dx} - EI_{z} \beta_{6} \int_{0}^{l} {vv_{xxxx} dx} + \left( {c_{w} \beta_{3} l/(m_{1} + m_{2} )} \right. \\ & \left. { + \beta_{5} } \right)\int_{0}^{l} {\dot{y}w_{t} dx} + \left( {c_{w} \beta_{4} /(m_{1} + m_{2} ) - c_{w} \beta_{5} /\rho A} \right)\int_{0}^{l} {e_{y} w_{t} dx} \\ &+ \left( {c_{v} \beta_{7} l/m_{2} + \beta_{9} } \right)\int_{0}^{l} {\dot{z}v_{t} dx} + \left( {c_{v} \beta_{8} /m_{2} - c_{v} \beta_{9} /\rho A} \right)\int_{0}^{l} {e_{z} v_{t} dx} \\ &+ \left( {\beta_{3} c_{w} /\rho A - \rho A\beta_{1} } \right)\int_{0}^{l} {w\ddot{y}dx} + \left( {\beta_{7} c_{v} /\rho A - \rho A\beta_{6} } \right)\int_{0}^{l} {v\ddot{z}dx} \\ &+ \beta_{3} \int_{0}^{l} {\ddot{y}w_{t} dx} + \beta_{7} \int_{0}^{l} {\ddot{z}v_{t} dx} + \left( {\beta_{3} l/(m_{1} + m_{2} )} \right)\dot{y}f_{y} \\ &+ \left( {\beta_{4} /(m_{1} + m_{2} )} \right)e_{y} f_{y} + \left( {\beta_{7} l/m_{2} } \right)\dot{z}f_{z} + \left( {\beta_{8} /m_{2} } \right)e_{z} f_{z} \\ &+ EI_{y} \left( {\beta_{3} /\rho A - \beta_{3} l/(m_{1} + m_{2} )} \right)\dot{y}w_{xxx} (0,t) + EI_{y} \left( {\beta_{5} /\rho A - \beta_{4} /(m_{1} + m_{2} )} \right)e_{y} w_{xxx} (0,t) \\ &+ EI_{z} \left( {\beta_{7} /\rho A - l\beta_{7} /m_{2} } \right)\dot{z}v_{xxx} (0,t) + EI_{z} \left( {\beta_{9} /\rho A - \beta_{8} /m_{2} } \right)e_{z} v_{xxx} (0,t). \\ \end{aligned}$$

According to (B.3) and (B.5), the time derivative of *V* is derived as follows.

B.6 $$\begin{aligned} \dot{V} &= \dot{y}\left[ {\left( {\beta_{3} l/(m_{1} + m_{2} ) + 1} \right)f_{y} + \left( {\beta_{4} + \beta_{5} l} \right)\dot{y}} \right. + EI_{y} \left( {\beta_{3} /\rho A} \right.\left. { - \beta_{3} l/(m_{1} + m_{2} )} \right)w_{xxx} (0,t)\left. { + \alpha_{1} e_{y} } \right] \\ &+ \dot{z}\left[ {\left( {\beta_{7} l/m_{2} + 1} \right)f_{z} } \right. + \left( {\beta_{8} + \beta_{9} l} \right)\dot{z}\left. { + EI_{z} \left( {\beta_{7} /\rho A - l\beta_{7} /m_{2} } \right)v_{xxx} (0,t) + \alpha_{3} e_{z} } \right] \\ &- \left[ {c_{w} \left( {1 + 2\alpha_{2} /\rho A} \right) - \rho A\beta_{1} } \right]\int_{0}^{l} {w_{t}^{2} dx} - \left[ {c_{u} \left( {1 + 2\alpha_{2} /\rho A} \right)} \right.\left. { - \rho A\beta_{2} } \right]\int_{0}^{l} {u_{t}^{2} dx} \\ &- \left[ {c_{v} \left( {1 + 2\alpha_{2} /\rho A} \right) - \rho A\beta_{6} } \right]\int_{0}^{l} {v_{t}^{2} dx} \\ &+ \beta_{1} \int_{0}^{l} {w\left( {Pw_{x} } \right)_{x} dx} + \beta_{1} \int_{0}^{l} {w\left( {EA\varepsilon w_{x} } \right)_{x} dx} - EI_{y} \beta_{1} \int_{0}^{l} {ww_{xxxx} dx} + \beta_{2} \int_{0}^{l} {EAu\varepsilon_{x} dx} \\ &+ \beta_{6} \int_{0}^{l} {v\left( {Pv_{x} } \right)_{x} dx} + \beta_{6} \int_{0}^{l} {v\left( {EA\varepsilon v_{x} } \right)_{x} dx} - EI_{z} \beta_{6} \int_{0}^{l} {vv_{xxxx} dx} \\ &+ \left( {\beta_{3} c_{w} /\rho A - \rho A\beta_{1} } \right)\int_{0}^{l} {w\ddot{y}dx} + \left( {c_{w} \beta_{3} l/(m_{1} + m_{2} ) + \beta_{5} } \right)\int_{0}^{l} {\dot{y}w_{t} dx} \\ &+ \left( {\beta_{7} c_{v} /\rho A} \right.\left. { - \rho A\beta_{6} } \right)\int_{0}^{l} {v\ddot{z}dx} + \left( {\beta_{7} lc_{v} /m_{2} } \right.\left. { + \beta_{9} } \right)\int_{0}^{l} {\dot{z}v_{t} dx} \\ &- c_{w} \left( {\beta_{5} /\rho A - \beta_{4} /(m_{1} + m_{2} } \right)\int_{0}^{l} {e_{y} w_{t} dx} - c_{v} \left( {\beta_{9} /\rho A - \beta_{8} /m_{2} } \right)\int_{0}^{l} {e_{z} v_{t} dx} \\ &+ \left( {\beta_{3} - 2\alpha_{2} } \right)\ddot{y}\int_{0}^{l} {w_{t} dx} + EI_{y} \left( {\beta_{5} /\rho A} \right.\left. { - \beta_{4} /(m_{1} + m_{2} )} \right)e_{y} w_{xxx} (0,t) + \left( {\beta_{4} /(m_{1} + m_{2} )} \right)e_{y} f_{y} \\ &+ \left( {\beta_{7} - 2\alpha_{2} } \right)\ddot{z}\int_{0}^{l} {v_{t} dx} + EI_{z} \left( {\beta_{9} /\rho A - \beta_{8} /m_{2} } \right)e_{z} v_{xxx} (0,t) + (\beta_{8} /m_{2} )e_{z} f_{z} . \\ \end{aligned}$$

By using integration by parts and Lemma 1, the following inequality and equation are obtained.

B.7 $$\begin{aligned} \beta_{1}\int_{0}^{l} {w\left( {Pw_{x} } \right)_{x} dx} &+ \beta_{1} \int_{0}^{l} {w\left( {EA\varepsilon w_{x} } \right)_{x} dx}+ \beta_{2} \int_{0}^{l} {u\left( {EA\varepsilon u_{x} } \right)_{x} dx}\\&+\beta_{6}\int_{0}^{l} {v\left( {Pv_{x} } \right)_{x} dx}+\beta_{6} \int_{0}^{l} {v\left( {EA\varepsilon v_{x} } \right)_{x} dx} \\ & \le - \beta_{1} \int_{0}^{l} {Pw_{x}^{2} dx} - \beta_{6} \int_{0}^{l} {Pv_{x}^{2} dx} + \delta_{5} EAl^{2} \left( {\int_{0}^{l} {w_{xx}^{2} dx} + \int_{0}^{l} {v_{xx}^{2} dx} } \right) \\ &- \left( {\beta_{2} - \left( {(2\beta_{1} - \beta_{2} )^{2} + (2\beta_{6} - \beta_{2} )^{2} } \right)/4\delta_{5} } \right)EA\int_{0}^{l} {\varepsilon^{2} dx,} \\ \end{aligned}$$

B.8 $$- \beta_{1} EI_{y} \int_{0}^{l} {ww_{xxxx} dx} = - \beta_{1} EI_{y} \int_{0}^{l} {w_{xx}^{2} dx} ; \, - EI_{z} \beta_{6} \int_{0}^{l} {vv_{xxxx} dx} = - \beta_{6} EI_{z} \int_{0}^{l} {v_{xx}^{2} dx}$$

where *δ*_5_ is a positive constant. It is noted that the inequalities $w_{x}^{4} \ll w_{x}^{2}$ and $v_{x}^{4} \ll v_{x}^{2}$ are used in (B.7). By letting *β*_*i*_ satisfy the following conditions

B.9 $$\beta_{3} = \beta_{7} = 2\alpha_{2} \beta_{3} c_{{\text{w}}} /\rho A = \rho A\beta_{1}, \, \beta_{7} c_{{\text{v}}} /\rho A = \rho A\beta_{6} , \, \beta_{5} /\rho A - \beta_{4} /(m_{1} + m_{2} ) \ge 0, \, \beta_{9} /\rho A - \beta_{8} /m_{2} \ge 0 $$

and using Lemmas 1 and 2 for the terms $\int_{0}^{l} {\dot{y}w_{t} dx}$, $\int_{0}^{l} {e_{{\text{y}}} w_{t} dx}$, $\int_{0}^{l} {\dot{z}v_{t} dx}$, and $\int_{0}^{l} {e_{{\text{z}}} v_{t} dx}$ of (B.6), we obtain

B.10 $$\begin{aligned} \dot{V} & \le - \left[ {c_{w} \left( {1 + 2\alpha_{2} /\rho A} \right) - \rho A\beta_{1} - c_{w} \delta_{7} \left( {\beta_{5} /\rho A\left. { - \beta_{4} /(m_{1} + m_{2} )} \right)} \right.} \right.\left. { - \delta_{6} \left( {c_{w} \beta_{3} l/(m_{1} + m_{2} ) + \beta_{5} } \right)} \right]\int_{0}^{l} {w_{t}^{2} dx} \\ &- \left[ {c_{u} \left( {1 + 2\alpha_{2} /\rho A} \right)} \right.\left. { - \rho A\beta_{2} } \right]\int_{0}^{l} {u_{t}^{2} dx} \\ &- \left[ {c_{v} \left( {1 + 2\alpha_{2} /\rho A} \right)} \right. - \rho A\beta_{6} - \delta_{9} c_{v} \left( {\beta_{9} /\rho A} \right.\left. { - \beta_{8} /m_{2} } \right)\left. { - \delta_{8} \left( {\beta_{7} lc_{v} /m_{2} + \beta_{9} } \right)} \right]\int_{0}^{l} {v_{t}^{2} dx} \\ &- \left( {\beta_{1} EI_{y} } \right.\left. { - \delta_{5} EAl^{2} } \right)\int_{0}^{l} {w_{xx}^{2} dx} - \left( {\beta_{6} EI_{z} - \delta_{5} EAl^{2} } \right)\int_{0}^{l} {v_{xx}^{2} dx} \\ &- \max \left( {\beta_{1} ,\beta_{6} } \right)\int_{0}^{l} {P\left( {w_{x}^{2} + v_{x}^{2} } \right)dx} - \left( {\beta_{2} - \left( {(2\beta_{1} - \beta_{2} )^{2} + (2\beta_{6} - \beta_{2} )^{2} } \right)/4\delta_{5} } \right)\int_{0}^{l} {EA\varepsilon^{2} dx} + D_{y} + D_{z} \\ \end{aligned}$$

where

B.11 $$\begin{aligned} D_{y} &= \left[ {\left( {\beta_{3} l/(m_{1} + m_{2} ) + 1} \right)f_{y} } \right. + \left( {\left( {c_{w} \beta_{3} l/(m_{1} + m_{2} ) + \beta_{5} } \right)l/\delta_{6} } \right.\left. { + \beta_{4} + \beta_{5} l} \right)\dot{y} \\ &+ EI_{y} \left( {\beta_{3} /\rho A - \beta_{3} l/(m_{1} + m_{2} )} \right)w_{xxx} (0,t) + \left. {\alpha_{1} e_{y} } \right]\dot{y} \\ &+ \left( {c_{w} l\left( {\beta_{5} /\rho A - \beta_{4} /(m_{1} + m_{2} )} \right)/\delta_{7} } \right)e_{y}^{2} + EI_{y} \left( {\beta_{5} /\rho A - \beta_{4} /(m_{1} + m_{2} )} \right)e_{y} w_{xxx} (0,t) \\ &+ \left( {\beta_{4} /(m_{1} + m_{2} )} \right)e_{y} f_{y} , \\ \end{aligned}$$

B.12 $$\begin{aligned} D_{z} &= \left[ {\left( {\beta_{7} l/m_{2} + 1} \right)f_{z} + \left( {\beta_{8} + \beta_{9} l + \left( {\beta_{7} lc_{v} /m_{2} + \beta_{9} } \right)l/\delta_{8} } \right)\dot{z}} \right. \\ & \left. { + EI_{z} \left( {\beta_{7} /\rho A - \beta_{7} l/m_{2} } \right)v_{xxx} (0,t) + \alpha_{3} e_{z} } \right]\dot{z} \\ &+ \left( {c_{v} l\left( {\beta_{9} /\rho A - \beta_{8} /m_{2} } \right)/\delta_{9} } \right)e_{z}^{2} + EI_{z} \left( {\beta_{9} /\rho A - \beta_{8} /m_{2} } \right)e_{z} v_{xxx} (0,t) + \left( {\beta_{8} /m_{2} } \right)e_{z} f_{z} . \\ \end{aligned}$$

In (B.10–B.12), *δ*_*i*_ (*i* = 6, 7, 8, 9) are positive constants. Substituting the control laws in (15) and (16) into (B.11) and (B.12), respectively, yields

B.13 $$\begin{aligned} D_{y} &= - \left[ {k_{1} - \beta_{4} - \beta_{5} l - \left( {c_{w} \beta_{3} l/(m_{1} + m_{2} ) + \beta_{5} } \right)l/\delta_{6} } \right]\dot{y}^{2} \\ &- \left[ {k_{2} \beta_{4} /(\beta_{3} l + m_{1} + m_{2} ) - c_{w} l\left( {\beta_{5} /\rho A} \right.} \right.\left. {\left. { - \beta_{4} /(m_{1} + m_{2} )} \right)/\delta_{7} } \right]e_{y}^{2} \\ &+ \left( {\beta_{3} EI_{y} /\rho A - \beta_{3} lEI_{y} /(m_{1} + m_{2} )} \right.\left. { - k_{3} } \right)\dot{y}w_{xxx} (0,t) + \left( {\alpha_{1} - k_{2} - k_{1} \beta_{4} /(\beta_{3} l + m_{1} + m_{2} )} \right)e_{y} \dot{y} \\ &+ \left( {\beta_{5} EI_{y} /\rho A - \beta_{4} EI_{y} /(m_{1} + m_{2} )} \right.\left. { - k_{3} \beta_{4} /(\beta_{3} l + m_{1} + m_{2} )} \right)e_{y} w_{xxx} (0,t), \\ \end{aligned}$$

B.14 $$\begin{aligned} D_{z} &= - \left[ {k_{4} - \beta_{8} - \beta_{9} l - \left( {\beta_{7} lc_{v} /m_{2} + \beta_{9} } \right)l/\delta_{8} } \right]\dot{z}^{2} \\ &- \left[ {k_{5} \beta_{8} /(\beta_{7} l + m_{2} ) - c_{v} l\left( {\beta_{9} /\rho A - \beta_{8} /m_{2} } \right)/\delta_{9} } \right]e_{z}^{2} \\ &+ \left( {\beta_{7} EI_{z} /\rho A - \beta_{7} lEI_{z} /m_{2} - k_{6} } \right)\dot{z}v_{xxx} (0,t) + \left( {\alpha_{3} - k_{5} - k_{4} \beta_{8} /(\beta_{7} l + m_{2} )} \right)e_{z} \dot{z} \\ &+ \left( {\beta_{9} EI_{z} /\rho A} \right.\left. { - \beta_{8} EI_{z} /m_{2} - k_{6} \beta_{8} /(\beta_{7} l + m_{2} )} \right)e_{z} v_{xxx} (0,t) \\ \end{aligned}$$

where

B.15 $$k_{i} = \left( {\beta_{3} l/(m_{1} + m_{2} ) + 1} \right)K_{i}, \,\, i = 1,2,3,$$

B.16 $$k_{j} = \left( {\beta_{7} l/m_{2} + 1} \right)K_{j}, \,\, j = 4,5,6.$$

If the following conditions hold

B.17 $$EI_{{\text{y}}} \left( {\beta_{3} /\rho A - \beta_{3} l/(m_{1} + m_{2} )} \right) - k_{3} = 0,$$

B.18 $$\alpha_{1} - k_{2} - k_{1} \beta_{4} /(\beta_{3} l + m_{1} + m_{2} ) = 0,$$

B.19 $$EI_{{\text{z}}} \left( {\beta_{9} /\rho A - \beta_{8} /m_{2} } \right) - k_{6} \beta_{8} /(\beta_{7} l + m_{2} ) = 0,$$

B.20 $$EI_{{\text{z}}} \left( {\beta_{7} /\rho A - \beta_{7} l/m_{2} } \right) - k_{6} = 0,$$

B.21 $$\alpha_{3} - k_{5} - k_{4} \beta_{8} /(\beta_{7} l + m_{2} ) = 0,$$

B.22 $$EI_{{\text{y}}} \left( {\beta_{5} /\rho A - \beta_{4} /(m_{1} + m_{2} )} \right) - k_{3} \beta_{4} /(\beta_{3} l + m_{1} + m_{2} ) = 0$$

then (B.10) can be rewritten as follows.

B.23 $$\begin{aligned} \dot{V} &\le - \left[ {k_{1} - \beta_{4} - \beta_{5} l - \left( {c_{w} \beta_{3} l/(m_{1} + m_{2} ) + \beta_{5} } \right)l/\delta_{6} } \right]\dot{y}^{2} \\ &- \left[ {k_{4} - \beta_{8} } \right.\left. { - \beta_{9} l - \left( {\beta_{7} lc_{v} /m_{2} + \beta_{9} } \right)l/\delta_{8} } \right]\dot{z}^{2} \\ &- \left[ {k_{2} \beta_{4} /(\beta_{3} l + m_{1} + m_{2} )} \right.\left. { - c_{w} l\left( {\beta_{5} /\rho A - \beta_{4} /(m_{1} + m_{2} )} \right)/\delta_{7} } \right]e_{y}^{2} \\ &- \left[ {k_{5} \beta_{8} /(\beta_{7} l + m_{2} )} \right.\left. { - c_{v} l\left( {\beta_{9} /\rho A - \beta_{8} /m_{2} } \right)/\delta_{9} } \right]e_{z}^{2} \\ &- \left[ {c_{w} \left( {1 + 2\alpha_{2} /\rho A} \right) - \rho A\beta_{1} } \right. - \delta_{6} \left( {c_{w} \beta_{3} l/(m_{1} + m_{2} ) + \beta_{5} } \right) - c_{w} \delta_{7} \left( {\beta_{5} /\rho A} \right.\left. {\left. { - \beta_{4} /(m_{1} + m_{2} )} \right)} \right]\int_{0}^{l} {w_{t}^{2} dx} \\ &- \left[ {c_{v} \left( {1 + 2\alpha_{2} /\rho A} \right) - \rho A\beta_{6} } \right.\left. {\left. { - \delta_{8} \left( {c_{v} \beta_{7} l/m_{2} + \beta_{9} } \right) - \delta_{9} c_{v} \left( {\beta_{9} /\rho A} \right. - \beta_{8} /m_{2} } \right)} \right]\int_{0}^{l} {v_{t}^{2} dx} \\ &- \left[ {c_{u} \left( {1 + 2\alpha_{2} /\rho A} \right) - \rho A\beta_{2} } \right]\int_{0}^{l} {u_{t}^{2} dx} - E\left( {\beta_{1} I_{y} - \delta_{5} Al^{2} } \right)\int_{0}^{l} {w_{xx}^{2} dx} - E\left( {\beta_{6} I_{z} - \delta_{5} Al^{2} } \right)\int_{0}^{l} {v_{xx}^{2} dx} \\ &- \max \left( {\beta_{1} ,\beta_{6} } \right)\int_{0}^{l} {P(w_{x}^{2} + v_{x}^{2} )dx} - \left( {\beta_{2} - \left( {(2\beta_{1} - \beta_{2} )^{2} + (2\beta_{6} - \beta_{2} )^{2} /4\delta_{5} } \right)} \right)\int_{0}^{l} {EA\varepsilon^{2} dx} . \\ \end{aligned}$$

Inequality (B.23) leads to the following result

B.24 $$\dot{V} \le - \lambda_{3} W_{2}$$

where $\lambda_{3} = \min \left( {\lambda_{31} ,\lambda_{32} ,...,\lambda_{311} } \right)$ and coefficients $\alpha_{i}$, $\beta_{j}$, and $\delta_{k}$ (*i* = 1, 2,…, 6, *j* = 1, 2,…,9, and *k* = 1, 2,…,8) satisfy the conditions:

B.25 $$\lambda_{31} = k_{1} - \beta_{4} - \beta_{5} l - \left( {c_{w} \beta_{3} l/(m_{1} + m_{2} ) + \beta_{5} } \right)l/\delta_{6} \ge 0,$$

B.26 $$\lambda_{32} = k_{2} \beta_{4} /(\beta_{3} l + m_{1} + m_{2} ) - c_{w} \left( {\beta_{5} /\rho A - \beta_{4} /(m_{1} + m_{2} )} \right)l/\delta_{7} \ge 0,$$

B.27 $$\lambda_{33} = \max \left( {\beta_{1} ,\beta_{6} } \right) \ge 0,$$

B.28 $$\lambda_{34} = k_{4} - \beta_{8} - \beta_{9} l - l\left( {c_{v} \beta_{7} l/m_{2} + \beta_{9} } \right)/\delta_{8} \ge 0,$$

B.29 $$\lambda_{35} = k_{5} \beta_{8} /(\beta_{7} l + m_{2} ) - c_{v} l\left( {\beta_{9} /\rho A - \beta_{8} /m_{2} } \right)/\delta_{9} \ge 0,$$

B.30 $$\lambda_{36} = c_{u} \left( {1 + 2\alpha_{2} /\rho A} \right) - \rho A\beta_{2} \ge 0,$$

B.31 $$\lambda_{37} = c_{w} \left( {1 + 2\alpha_{2} /\rho A} \right) - \rho A\beta_{1} - \delta_{6} \left( {c_{w} \beta_{3} l/(m_{1} + m_{2} ) + \beta_{5} } \right) - c_{w} \delta_{7} \left( {\beta_{5} /\rho A - \beta_{4} /(m_{1} + m_{2} } \right) \ge 0,$$

B.32 $$\lambda_{38} = c_{v} \left( {1 + 2\alpha_{2} /\rho A} \right) - \rho A\beta_{6} - \delta_{8} \left( {c_{v} \beta_{7} l/m_{2} + \beta_{9} } \right) - \delta_{9} c_{v} \left( {\beta_{9} /\rho A - \beta_{8} /m_{2} } \right) \ge 0,$$

B.33 $$\lambda_{39} = E\left( {\beta_{1} I_{y} - \delta_{5} Al^{2} } \right) \ge 0,$$

B.34 $$\lambda_{310} = E\left( {\beta_{6} I_{z} - \delta_{5} Al^{2} } \right) \ge 0,$$

B.35 $$\lambda_{311} = \beta_{2} - \left( {(2\beta_{1} - \beta_{2} )^{2} + (2\beta_{6} - \beta_{2} )^{2} } \right)/4\delta_{5} \ge 0.$$

Based on Lemma 4, the following inequality is derived

B.36 $$\dot{V} \le - \lambda_{3} W_{2} \le - (\lambda_{3} /\lambda_{2} )V \Rightarrow \dot{V} \le - \lambda V$$

where $\lambda = \lambda_{3} /\lambda_{2}$. Accordingly, Lemma 5 is proven.
